# O4.2. HERITABILITY AND CORRELATION TO SCHIZOPHRENIA SPECTRUM DISORDER OF GLUTAMATE AND OTHER NEUROMETABOLITE LEVELS IN ANTERIOR CINGULATE AND LEFT THALAMUS: A REGISTER BASED MAGNETIC RESONANCE TWIN STUDY

**DOI:** 10.1093/schbul/sby015.208

**Published:** 2018-04-01

**Authors:** Christian Legind, Brian Broberg, Rene Mandl, Rachel Brouwer, Simon Anhøj, Rikke Hilker, Maria H Jensen, Philip McGuire, Hilleke E Hulshoff Pol, Birgitte Fagerlund, Egill Rostrup, Birte Glenthoj

**Affiliations:** 1 Psychiatric Center Glostrup; 2 Center for Neuropsychiatric Schizophrenia Research; 3 University Medical Center Utrecht; 4 Center for Neuropsychiatric Schizophrenia Research and Center for Clinical Intervention and Neuropsychiatric Schizophrenia Research, Copenhagen University Hospital, Mental Health Center Glostrup; 5 Institute of Psychiatry, Psychology & Neuroscience, King’s College London; 6 Brain Center Rudolf Magnus, University Medical Center Utrecht; 7 Center for Neuropsychiatric Schizophrenia Research, Psychiatric Center Glostrup, Mental Health Services Capital Region of Denmark, University of Copenhagen and The Lundbeck Foundation Initiative for Integrative Psychiatric Research (iPSYCH); 8 Center for Neuropsychiatric Schizophrenia Research, & Center for Clinical Intervention and Neuropsychiatric Schizophrenia Research, Psychiatric Center Glostrup, University of Copenhagen, & Glostrup University Hospital

## Abstract

**Background:**

Glutamatergic changes in schizophrenia may precede dopaminergic alterations leading to psychopathology as part of a dopaminergic pathway, or they may represent a distinct pathophysiology. It is unknown if these glutamatergic alterations are due to genetic influences, although findings in gene studies implicate the NMDA receptor. Higher levels of glutamate and glutamine have been reported in the thalamus of patients with schizophrenia, while studies of the anterior cingulate cortex (ACC) have reported increased, decreased, and unaltered levels compared to healthy controls.

By studying discordant mono- (MZ) and dizygotic (DZ) twins it is possible to estimate heritability and correlation to disease in the same population. This kind of study has only been done once on glutamate levels, but no heritability estimates were reported.1 A study of older healthy twins found N-acetyl aspartate (NAA), choline (Cho), creatinine (Cr), and myo-inositol (MI) levels to be heritable in posterior cingulate cortex.2 Of these metabolites, NAA has generally been found to correlate negatively with schizophrenia. Here we present our final results on heritability, and correlation to liability for schizophrenia spectrum disorder (ICD-10 F2x.x) of the neurometabolite levels in the ACC and the left thalamus.

**Methods:**

By linking The Danish Twin Register and The Danish Psychiatric Central Research Register, 25 complete MZ and 21 complete DZ twin pairs con- or discordant for schizophrenia spectrum disorder (ICD 10 F2x.x) and 29 complete MZ and 20 complete DZ healthy control pairs were included. Thirteen additional twins were scanned without their siblings. Spectra of glutamate, Glx, NAA, Cho, Cr and MI were obtained by [1H]-MR spectroscopy at 3 tesla and analyzed by using LCModel. Additive genetic, common environmental and unique environmental effects on metabolite levels were calculated by structural equation modeling with openMX software. The best fitting model was determined by the Akaike Information Criterion.

**Results:**

In the ACC heritability estimates were significant for glutamate (29%), Glx (31%), NAA (39%), Cho (38%), Cr (37%) and MI (33%). In the left thalamus we found significant estimates of heritability for glutamate (16%), Glx (31%), Cho (60%), and of common environment for Cr (29%). A significant positive correlation to schizophrenia spectrum liability was found for glutamate in the left thalamus (r=0.16; p = 0.03), and negative correlations were found for NAA (r = -0.16; p = 0.02) and Cr (r = -0.25; p = 0.006) in the ACC. For glutamate in the thalamus and Cr in the ACC the significant correlation to disease was due to overlapping genetic effects influencing both metabolite and disease.

**Discussion:**

In this the first study to estimate heritability of glutamate levels in the brain, the primary findings are that glutamate levels in both the ACC and the left thalamus are heritable, and in the left thalamus also correlated to disease with a significant genetic overlap. This emphasizes glutamate levels in the left thalamus as a potential endophenotypic marker for schizophrenia. NAA and Cr were negatively correlated to disease in the ACC, which could point to disturbances of neuronal health and metabolism. For Cr an overlap of genes influencing both metabolite levels and disease suggests Cr as a possible candidate endophenotype.

**References:**

1. Lutkenhoff ES, van Erp TG, Thomas M a, Therman S, Manninen M, Huttunen MO et al. Proton MRS in twin pairs discordant for schizophrenia. Mol Psychiatry 2010; 15: 308–18.

2. Batouli SAH, Sachdev PS, Wen W, Wright MJ, Suo C, Ames D et al. The heritability of brain metabolites on proton magnetic resonance spectroscopy in older individuals. Neuroimage 2012; 62: 281–289.

